# Post-traumatic growth experience of breast cancer patients: A qualitative systematic review and meta-synthesis

**DOI:** 10.1371/journal.pone.0316108

**Published:** 2025-01-23

**Authors:** ShiNi Huang, Min Huang, Fang Long, Fang Wang

**Affiliations:** 1 Department of Nursing, Chengdu University of Traditional Chinese Medicine, Chengdu, Sichuan Province, China; 2 Department of Basic Medicine, Chengdu University of Traditional Chinese Medicine, Chengdu, Sichuan Province, China; 3 Guang’an Hospital of Traditional Chinese Medicine, Guang’an, Sichuan Province, China; The Hong Kong Polytechnic University, HONG KONG

## Abstract

**Objectives:**

This study aimed to systematically incorporate the post-traumatic growth experience of breast cancer patients and furnish insights for the formulation of targeted psychological care measures.

**Methods:**

The search period we were ranged from establishing the database to February 2024. We systematically searched four Chinese databases and seven English databases. The focus was on collecting qualitative research literature regarding the post-traumatic growth experience of breast cancer patients. Literature was screened and analyzed using Endnote20 software. Quality evaluation was conducted using the authenticity evaluation criteria (2016 edition) recommended by the JBI. The results were synthesized utilizing the meta-integration methodology established by the Joanna Briggs Institute (JBI) and adhered to the reporting standards delineated in the PRISMA checklist, as well as the ENTREQ reporting guidelines.

**Results:**

This study encompassed 11 studies, from which 42 themes and 68 sub-themes were extracted. Similar research findings were categorized into 11 new classifications, leading to the formulation of four integrative conclusions: self-reconstruction, enhancement of spirituality and religious beliefs, appreciation of the new philosophy of life, and transformations associated with others.

**Conclusions:**

Medical professionals should possess a precise comprehension of the concept of post-traumatic growth, and actively encourage patients to engage in deliberate rumination, facilitate emotional release, support effective coping strategies, enhance social support systems, foster post-traumatic growth in patients, and promote their overall well-being.

**Trial registration:**

Systematic review registration:

CRD42024519850.

## 1. Introduction

Breast cancer ranks as the most prevalent malignant tumor affecting women. According to global cancer data statistics [1], breast cancer outstripped lung cancer as the top cause of worldwide cancer incidence in 2020, accounting for 11.7% of all cases during that year. The pathogenesis of breast cancer is intricate and involves multiple cellular types and processes [2]. Currently, recommended primary treatment modalities encompass surgical intervention and systemic therapy (including endocrine therapy, chemotherapy, and targeted therapy). With an increasing number of individuals surviving cancer, greater attention has been directed towards the psychological impact resulting from diagnosis and subsequent treatments. Cancer significantly impacts patients’ physical and mental well-being and is considered a traumatic experience for affected individuals [3]. It may provide an opportunity for post-traumatic growth (PTG), which exhibits a robust positive correlation with traumatic experiences, particularly within the context of breast cancer. Breast cancer survivors exhibit higher PTG values compared to those with oral cavity or throat cancers or patients undergoing rehabilitation [4].

Originally brought forward by Tedeschi and Calhoun in 1996 [5], Post-Traumatic Growth (PTG) is the prevailing model utilized to elucidate positive psychological transformations in cancer patients following their illness. It pertains to the constructive psychological changes experienced due to grappling with an exceedingly arduous life circumstance rather than alterations caused directly by the event itself. The most frequently employed assessment tool is the Post-Traumatic Growth Scale (PTGI), which evaluates five primary domains: (1) a profound understanding of life and a transfer of consciousness in priorities; (2) fostering of deeper and closer interpersonal connections;(3) have a more vigorous perception of personal power; (4) get acquainted with new possibilities or new routes in life; and (5) spiritual advancement.

Evidence supports the presence of PTG in individuals diagnosed with cancer [3,6], most commonly reported within three years following diagnosis [7]. This phenomenon yields physical and psychological benefits for patients while associated with positive health behaviors, including improved dietary habits, increased physical activity levels, and heightened workplace safety awareness [8]. Furthermore, PTG demonstrates positive correlations with enhanced recovery rates for white blood cell counts post-chemotherapy [9] alongside cortisol’s circadian rhythm [10]—suggesting a connection exists between PTG and decreased stress levels and healthier endocrine functions. Higher levels of PTG correspond to increased patient survival rates [9]. Casellas-Grau et al.’s findings [11] reveal an inverse relationship between PTG and depressive/anxious symptoms while demonstrating direct associations with hopefulness, optimism, spirituality, and sense-making. Meta-analytical outcomes suggest that those experiencing PTG are more likely to adapt to their condition effectively, report superior subjective well-being across both physical and mental domains, experience milder pain symptoms along with trauma-induced stress responses, engage in healthier lifestyle choices, enjoy elevated quality-of-life standards, and exhibit greater adherence towards their treatment regimens [12,13]. However, PTG is distinct from benefit finding, as they are related but not synonymous [14]. Benefit finding may be transient, whereas PTG has the potential to impact a patient’s life [15] significantly. It is worthy of emphasis that not all cancer patients are capable of perceiving the positive effect of PTG, and that specific individuals diagnosed with cancer might still confront the negative consequences attributed to post-traumatic stress disorder (PTSD) [14].

The existing literature on PTG in both women and men reveals significant gender differences, with women exhibiting higher levels of PTG compared to men who have encountered similar traumatic experiences [16]. These findings align with previous research [17–19]. Female patients are more inclined than male patients to perceive life stressors as threats [3,19], given that PTG can only emerge following the confrontation of life events deemed threatening. Consequently, female cancer patients are more likely to experience PTG compared to their male counterparts.

The existing studies have predominantly focused on intervention measures, influential factors, and predictors of PTG in BC patients. However, they have not fully captured the authentic inner experiences of these patients. In contrast to quantitative research, qualitative research offers a more accurate reflection of the genuine psychological changes and emotional experiences related to PTG in BC survivors. While a scoping review has been conducted outlining cancer patients’ experiences with PTG, it primarily delves into the scope and nature of qualitative research on PTG rather than specific patient experiences. Although recent qualitative studies on PTG among breast cancer patients have increased, these experiences are subjective and individualistic. A single qualitative study may not comprehensively represent the situation, and its clinical implications are limited. Therefore, this study adopts a meta-integrative approach to summarize and analyze relevant findings from qualitative research. By uncovering genuine PTG experiences in breast cancer patients, this study aims to analyze positive psychological changes and emotional experiences associated with PTG in order to inform the development of pertinent psychological nursing interventions.

## 2. Materials and methods

### 2.1 Methodological guidelines

Meta-aggregation is a systematic review method in qualitative research that employs inductive reasoning to analyze, classify, and synthesize the findings of qualitative studies [20]. This approach emphasizes the integration of multiple qualitative results, leading to the development of cohesive concepts and providing comprehensive explanations and integrative meanings. It constitutes a dynamic and iterative process of interpretation and reflection [21]. This study employed the JBI meta-aggregation method to facilitate data extraction and synthesis [22]. This method is grounded in the philosophical traditions of pragmatism and Husserlian transcendental phenomenology [23]. This approach systematically reviews qualitative studies to generate integrated findings that inform healthcare practice or policy. These findings are presented as recommendations, offering generalizable statements to guide practitioners and decision-makers [24]. This is entirely consistent with the objectives of this study.

Consequently, the research employed the quality assessment criteria for qualitative studies established by JBI to evaluate the quality of the included literature while adhering to both PRISMA and ENTREQ reporting guidelines. The PRISMA checklist can be found in S1 File, and the ENTREQ reporting guidelines are available in S2 File. The study protocol has been registered on PROSPERO (CRD42024519850).

### 2.2 Data sources and search strategy

We systematically query four Chinese databases, including China Biomedical Literature Database (CBM), WanFang, China National Knowledge Infrastructure (CNKI), and VIP, alongside seven English databases such as PubMed, Embase, CINAHL, PsyINFO, Web of Science, SCOPUS, and Cochrane Library to comprehensively retrieve qualitative studies focusing on the PTG experiences of BC patients. The search encompasses data from the inception of these databases up to February 2024. Additionally, a thorough examination of the references cited in the included studies is conducted to supplement the acquisition of pertinent literature. The search strategy incorporates terms such as “breast neoplasms,"、" breast cancer,"、" post-traumatic growth, "、"posttraumatic growth,"、"ptg,"、" qualitative research,"、" qualitative study, “、” phenomenology,*”and so on. For further detailed information regarding the search strategies, please refer to S3 File.

### 2.3 Eligibility and screening of the literature

We established inclusion and exclusion criteria for the study based on research requirements. The following are the inclusion criteria presented:

1. Participants (P): patients without severe mental disorders, diagnosed with breast cancer, aged 18 or above, of any gender, with no restrictions on treatment methods or disease stage.
2. Phenomenon of interest (I): the trauma-informed growth experience of breast cancer patients during their life or treatment process.
3. Context (Co): patients in hospitals, families, or medical institutions.
4. Study design (S): qualitative studies or mixed-methods studies reporting qualitative results, with no restrictions on research methods such as grounded theory, ethnography, phenomenology, case studies, and action research. In the case of mixed-methods studies, only the qualitative part was analyzed.

The following are the exclusion criteria listed:

1. literatures that are neither in Chinese nor in English;
2. duplicated or unavailable full-text literature;
3. gray literature published in dissertations or newspapers without formal publication.
4. other trauma-informed growth experiences related to cancer.

We used Endnote 20 software to screen and analyze the literature by importing all citations and removing duplicates. Two reviewers independently screened articles based on title and abstract information, while any divergences were settled by a third reviewer. A second round of review was conducted for articles meeting the inclusion criteria using a process similar to the first round. The PRISMA flowchart for systematic review and meta-analysis according to preferred reporting items showed the results of literature screening.

### 2.4 Data extraction

Two reviewers were designated to extract data from both Chinese and English literature. The reviewer assigned to the Chinese literature translated the research findings into English, which the other two reviewers subsequently assessed to ensure that the translation accurately represented the original Chinese text. In instances of disagreement, they would consult a fourth reviewer until all reviewers reached a consensus. Two reviewers autonomously extracted data from the included studies utilizing standardized forms. In case of disagreement, they discussed the issue with a third reviewer. The essential features of the included studies, such as authors, year of publication, country, study design, study population and characteristics, interesting phenomena, and primary outcomes, were extracted using standardized forms.

### 2.5 Quality appraisal

The process involved two reviewers who independently assessed the quality of the included studies using the "Quality Appraisal Standards for Qualitative Research" from the Australian Centre for Evidence-Based Healthcare (JBI). Both reviewers had received training in evidence-based practice methodology. Each criterion was assessed with responses of "yes," "no," "unclear," or "not applicable." The incorporated studies were sorted into three levels: A, B, and C. Studies that fully met the quality standards were designated as level A, suggesting the existence of a low risk of bias; those that partially met the standards were classified as level B, suggesting the existence of a moderate risk of bias; and those that did not meet the standards were classified as level C, suggesting the existence of a high risk of bias. In cases where there was disagreement between reviewers’ assessments, a third reviewer provided input to facilitate consensus. Ultimately, only studies rated at levels A and B in terms of quality were included.

### 2.6 Data synthesis

This study employed the JBI meta-aggregation method to synthesize the research findings systematically (S4 File). In the initial stage, the reviewers exhaustively examined the included literature and categorized it according to its subject and content features, thereby forming a descriptive theme. Ultimately, the newly constructed themes underwent repeated scrutiny, refining new concepts, viewpoints, or hypotheses and culminating in final analysis results comprising themes and subthemes that offer novel explanations of phenomena and meanings, enhancing results’ reliability, specificity, and generalizability. Two researchers embarked on the initial content extraction and classification protocol, with one tasked with identifying novel descriptive and analytical themes. The entire research team then reviewed the data analysis procedure to guarantee consistency in the interpretation and appropriateness of analytical themes. All authors scrutinized the synthesized results, with any discrepancies resolved through discussion. All analyses were performed using Excel files.

### 2.7 ConQual-assessment of confidence of evidence

The ConQual system, established by JBI in 2014, evaluates and categorizes meta-synthesized evidence from qualitative studies [25]. This system assesses the credibility and dependability of integrated evidence, resulting in quality ratings classified as high, moderate, low, or very low according to ConQual criteria. Dependability is dissected based on five aspects for evaluating the quality of the original studies encompassed in the comprehensive outcomes. In comparison, credibility is appraised based on three aspects to ascertain whether the comprehensive results follow the supporting data. Two reviewers conducted the evaluation process; in cases where there was disagreement between reviewers’ assessments, a third reviewer provided input to facilitate consensus.

## 3. Results

### 3.1 Literature search results

**Fig 1:**
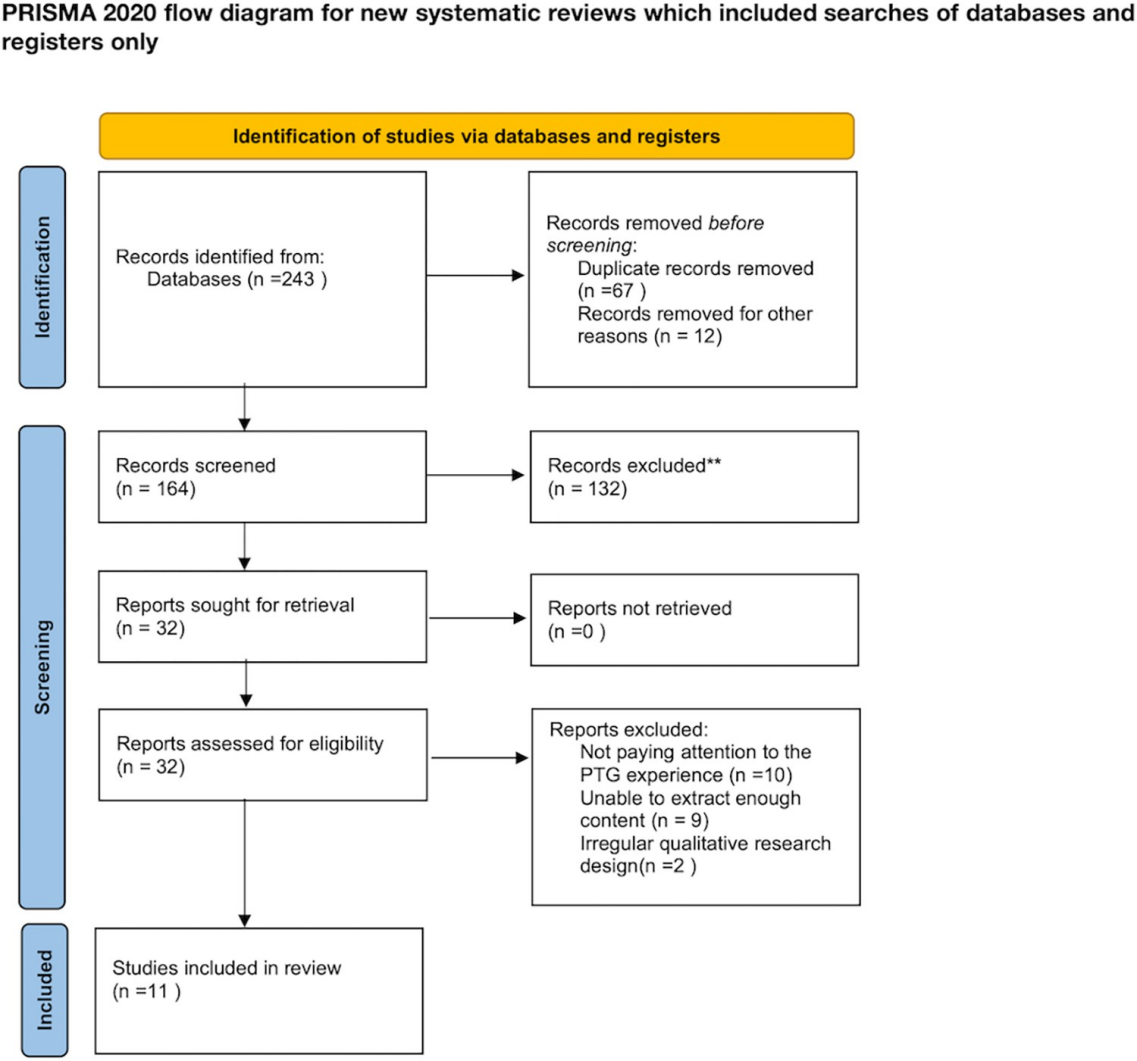
PRISMA flow diagram of literature screening.

Fig 1 depicts the procedure of literature screening. In total, 243 studies were initially retrieved from Chinese and English databases. After removing 67 duplicate studies, a review of titles and abstracts based on inclusion criteria excluded 132 studies. Subsequently, a thorough reading of the full text for the remaining 32 studies was conducted, followed by further evaluation to determine compliance with the inclusion criteria. Ultimately, 11 articles were included, comprising 4 in Chinese [26–29] and 7 in English [30–36].

### 3.2 Quality appraisal

The outcomes of the quality evaluation concerning all the incorporated studies are presented in Table 1. All studies were assigned a rating of Grade B. One study lacked clarity regarding the researcher’s cultural background and values, while the remaining did not offer detailed information. Additionally, one study was unclear about whether there was an impact on the researcher, with no mention of this issue in other studies. Furthermore, one study lacked clarity on whether there was consistency in methodology and data collection methods. All studies had obtained approval from the ethics committee.

**Table 1:** Quality appraisal of the included studies.

Last name of the first author et al. (publication year)	Is there congruity between the stated philosophical perspective and the research methodology?	Is there congruity between the research methodology and the research question or objectives?	Is there congruity between the research methodology and the methods used to collect data?	Is there congruity between the research methodology and the representation and analysis of data?	Is there congruity between the research methodology and the interpretation of results?	Is there a statement locating the researcher culturally or theoretically?	Is the influence of the researcher on the research, and vice-versa, addressed?	Are participants, and their voices, adequately represented?	Is the research ethical according to current criteria or, for recent studies, is there evidence of ethical approval by an appropriate body?	Do the conclusions drawn in the research report flow from the analysis, or interpretation, of the data?
Horgan et al. (2010) [30]	Y	Y	Y	Y	Y	N	N	Y	Y	Y
Li et al (2012) [29]	Y	Y	Y	Y	Y	N	N	Y	Y	Y
Fallah et al. (2012) [31]	Y	Y	U	Y	Y	N	N	Y	Y	Y
Tsuchiya et al. (2013) [32]	Y	Y	Y	Y	Y	U	N	Y	Y	Y
Mehrabi et al. (2015) [33]	Y	Y	Y	Y	Y	N	N	Y	Y	Y
Barthakur et al. (2016) [34]	Y	Y	Y	Y	Y	N	N	Y	Y	Y
Tang et al. (2019) [26]	Y	Y	Y	Y	Y	N	N	Y	Y	Y
İnan et al. (2020) [35]	Y	Y	Y	Y	Y	N	N	Y	Y	Y
Zhai et al (2021) [36]	Y	Y	Y	Y	Y	N	U	Y	Y	Y
Tan et al (2023) [27]	Y	Y	Y	Y	Y	N	N	Y	Y	Y
Yan et al. (2023) [28]	Y	Y	Y	Y	Y	N	N	Y	Y	Y

Abbreviations: Y: Yes, N: No, U: Uncertain.

### 3.3 Basic features of the literature

Table 2 unfolds the characteristics of all the incorporated studies, all publications from 2012 to 2023 (see Table 2). Sample sizes of the eleven incorporated studies varied between 10 and 24 individuals. One hundred sixty-nine participants were included in the study, and the participants’ demographic information is presented in Table 3. While five investigations took place within China’s borders [26–29,36], others occurred across diverse locations, including Iran [31,33], India [34], the UK [30], Turkey [35], and Japan [32] respectively. Predominantly hospital-based interviews characterized most of these inquiries with a single instance utilizing an open-ended questionnaire approach [31]. The female participants encompassed ages ranging from 29 to 75 years old. Five surveys employed purposive sampling techniques, whereas others utilized methodologies such as telephone or letter invitations. Descriptive phenomenological analysis emerged as a frequently adopted methodology closely followed by grounded theory.

**Table 2:** Characteristics of the incorporated studies.

Author(s) &Publication Year	Research location	Design (Methodology &data collection method)	Characteristics of study participants	Phenomena of interest	Main research findings	
Horgan et al. (2010) [30]	United Kingdom (England)	grounded theory、semi-structured interview	20 Women with breast cancer, age range 32–75 years, diagnosis time range from 3 months—28 years	To explore the PTG experience of BC patients	1. Change the focus of life	
					2. Increase empathy	
					3. Boosting confidence	
Li et al (2012) [29]	China	phenomenological approach、in-depth interviews	10 cases of female BC patients during chemotherapy following surgery, age range 31-66years	To explore the PTG of female BC patients during chemotherapy following surgery	1. The coexistence of negative psychology and awareness of fighting against adverse events	
					2. Embrace confidence in the fight against diseases	
					3. Start to re-examine life and health	
					4. Start planning for the future and actively respond	
Fallah et al. (2012) [31]	Iran	Interpretative phenomenology、open-ended questionnaire	23 female patients with breast cancer, between 34 and 61 years old	To explore the PTG experience of Iranian Women	1. spiritual growth	
					2. appreciation of life	
					3. increased personal strengths	
Tsuchiya et al. (2013) [32]	Japan	phenomenological approach、Semi-structured interview、focus group discussions	10 Japanese breast cancer survivors, age range 39–69 years	To investigate the PTG experience of Japanese breast cancer survivors after diagnosis	1. attitudinal changes towards life	
					2. strengthening trust in family and friends	
					3. increased appreciation of life	
					4. self-development	
					5.future perspectives	
					6.education for friends	
					7.efforts towards bodily change	
Mehrabi et al. (2015) [33]	Iran	qualitative phenomenological approach、semi-structured in-depth interviews	18 Iranian breast cancer survivors aged range 31–65 years	To explore the PTG experience of Iranian Women	1. appreciate of life	
					2. stability	
					3. spiritual prosperity	
					4. effective interaction	
Barthakur et al. (2016) [34]	India	Descriptive phenomenological approach、Semi-structured interview	15 Indian women with breast cancer, age range 45–72 years	To delve into the PTG experience of female BC survivors in India	1. Cancer: A new lease on life	
					2. Self: A priority and "stronger mentally."	
					3. Relationships: Closer, more empathetic, and generous	
					4. Spirituality	
Tang et al. (2019) [26]	China	phenomenological approach、in-depth interviews, and observation method	12 young and middle-aged BC survivors, age range 29–44 years	To probe into the experience of PTG of young and middle-aged survivors with breast cancer after surgery	1. Self-change	
					2. Sprouts of thinking transformation	
					3. Changes in relationships with others	
					4. Changes in the direction of life	
İnan et al. (2020) [35]	Turkey	descriptive phenomenological approach、Semi-structured interview	13 BC survivors	To describe the PTG experience of BC survivors in Turkey	1. making sense of cancer	
					2. positive restoring	
Zhai et al (2021) [36]	China	constructivist grounded theory approach、semi-structured in-depth interviews	24 Chinese participants, age range of 26 to 66 years	To describe the PTG experience of Chinese women in adapting to breast cancer	1. renewed self-perception	
					2. encountering changes in relationships	
					3. altering philosophical values and beliefs	
Tan et al (2023) [27]	China	phenomenological approach、Semi-structured interview	12 young patients with breast cancer, age range 30–45 years	To probe into the PTG experience of young patients	1. Personality change	
					2. Changes in relationships with others	
					3. Changes in life	
					4. Significance of illness	
					5. Gratitude and dedication	
Yan et al. (2023) [28]	China	phenomenological approach、semi-structured in-depth interviews	12 BC patients after breast reconstruction, age range 39–60 years	To understand the real PTG experience of patients with breast cancer after breast reconstruction	1. Self-reflection	
					2. Self reshaping	
					3. Self-realization	

**Table 3:** Demographic information of the participants.

Author(s) &Publication Year	Sample size	Therapeutic approach	The interval between diagnosis and follow-up time duration for each study	time duration for each study	Disease Stage
Horgan et al. (2010) [30]	20	All participants underwent surgical intervention. Additionally, 14 participants received chemotherapy, ten received radiotherapy, and 13 were administered adjuvant pharmacotherapy.	The mean duration from diagnosis is four years and eight months, ranging from 3 months to 28 years	June 2005 to January 2006	Stage 0-IV
Li et al (2012) [29]	10	Undergo surgical intervention followed by chemotherapy.	The study did not specify exact time points; instead, it documented the interval between participants’ involvement in the study and the surgical procedure, which ranged from 4 to 9 days.	August to October 2011	The study did not specify exact time points
Fallah et al. (2012) [31]	23	ll patients underwent surgical intervention, and except one individual, all received both chemotherapy and radiotherapy.	The duration following diagnosis varies from 8 to 55 months	From March 2010 to September 2010	Ten women are in Stage I of the disease, six women are in Stage II, and seven cases are in Stage III
Tsuchiya et al. (2013) [32]	10	Eight participants underwent mastectomy, while two underwent lumpectomy. Additionally, seven participants were receiving hormone therapy during the study	The study did not specify exact time points	The study did not specify exact time points	Nine participants were diagnosed with early-stage breast cancer (stages I and II), while one participant was diagnosed with advanced-stage breast cancer (stage III)
Mehrabi et al. (2015) [33]	18	All patients underwent surgical intervention and completed adjuvant therapy within 3 to 6 months prior to the initiation of the study	The study did not specify exact time points	he research was undertaken in 2014; the authors did not provide a specific conclusion date	The study did not specify exact time points
Barthakur et al. (2016) [34]	15	All patients underwent surgical intervention, with 11 receiving chemotherapy, 12 undergoing radiation therapy, and 9 receiving hormone adjuvant treatment	The duration of the study ranged from 3 to 28 years	January 2012 to August 2013	The study did not specify exact time points
Tang et al. (2019) [26]	12	All patients underwent surgical treatment, except for one patient who had a shorter duration since diagnosis and did not receive either chemotherapy or radiation therapy. Five patients received chemotherapy, while the remaining six were treated with both chemotherapy and radiation therapy	The duration of diagnosis spans from 3 months to 5 years and ten months	The study did not specify exact time points	The study did not specify exact time points
İnan et al. (2020) [35]	13	All patients underwent systemic therapy for breast cancer	The study did not specify exact time points	August 2015 to January 2016	Stage I-III
Zhai et al (2021) [36]	24	All participants underwent surgical intervention, and regarding adjuvant therapy, 17 women received chemotherapy alone, while the remaining participants were treated with both chemotherapy and radiation therapy	The interval between diagnosis and the commencement of the study varied from one year to 13 years	The study did not specify exact time points	The study did not specify exact time points
Tan et al (2023) [27]	12	All patients underwent surgical intervention and received combined chemotherapy and radiotherapy	The interval between diagnosis and the study initiation spans 9 to 24 months	October 2021 to March 2022	The study did not specify exact time points
Yan et al. (2023) [28]	12	All patients received breast reconstruction surgery	The study did not specify exact time points	April 2022 to July 2022	The study did not specify exact time points

### 3.4 ConQual-assessment of confidence of evidence

The dependability ratings for all studies included in the analysis predominantly scored 3. However, the levels of evidence within the qualitative studies were distinctly defined, and their credibility was upheld. Consequently, it was concluded that the ConQual level is moderate. The scores of the ConQual system for this review are depicted in Table 4.

**Table 4:** ConQual system scores for this review.

Synthesized findings	Type of research	Dependability	Credibility	ConQual score	Comments
self-reconstruction	Qualitative research -phenomenological,grounded theory descriptive—High	Downgrade one level Moderate*	Remains unchanged	Moderate**	The findings came from 11 papers
					*Downgraded one level as the majority of studies (10 out of 11) scored 3 on questions related to the appropriateness of the conduct of the study
					**Remains unchanged as all findings unequivocal
enhancement of spirituality and religious beliefs	Qualitative research-phenomenological,grounded theory descriptive—High	Downgrade one level Moderate*	Remains unchanged	Moderate	The findings came from 9 papers
					*Downgraded one level as the majority of studies (8 out of 9) scored 3 on questions related to the appropriateness of the conduct of the study
					**Remains unchanged as all findings unequivocal
appreciate the new philosophy of life	Qualitative research-phenomenological,grounded theory descriptive—High	Downgrade one level Moderate*	Remains unchanged	Moderate	The findings came from 9 papers
					*Downgraded one level as the majority of studies (8 out of 9) scored 3 on questions related to the appropriateness of the conduct of the study
					**Remains unchanged as all findings unequivocal
transformations associated with others	Qualitative research-phenomenological,grounded theory descriptive—High	Downgrade one level Moderate*	Remains unchanged	Moderate	The findings came from 11 papers
					*Downgraded one level as the majority of studies (10 out of 11) scored 3 on questions related to the appropriateness of the conduct of the study
					**Remains unchanged as all findings unequivocal

### 3.5 Review findings

Sixty-eight research findings were extracted from the 11 included studies. The research findings were synthesized using the JBI meta-aggregation method, enabling comparisons and contrasts to be drawn. This process formed 11 distinct categories, ultimately producing four synthesized research outcomes. The details of the synthesized research themes are outlined in Table 5.

**Table 5:** Synthesized research themes.

Synthesized findings	Category
Self-reconstruction	Cultivate a renewed self-awareness
	The enhancement of personal strength
	Engage in proactive self-management of health conditions
	Always remember to be thankful
Enhancement of spirituality and religious beliefs	Enhancing the inner self to facilitate spiritual development
	Reinforce one’s religious convictions
Appreciation of the new philosophy of life	Identify new opportunities and potential possibilities
	The life priorities have undergone a transformation
Transformations associated with others	Proactively engage in establishing connections with others
	Modification of interpersonal relationships
	By assisting others through individual actions

#### 3.5.1 Synthesized finding1: Self-reconstruction

BC patients often renew their understanding of themselves after experiencing physical and emotional challenges. Through introspection regarding their cancer journey and past experiences, they reevaluate the significance and positioning of their identity, resulting in the emergence of positive personal strength, an enhancement of self-confidence, the development of a new perspective on health, and a transformation in disease management behaviors. Additionally, they express gratitude to those who have supported them throughout their cancer journey.

*3*.*5*.*1*.*1Category1*: *Cultivate a renewed self-awareness*. In the early stages of diagnosis, patients frequently grapple with feelings of bewilderment regarding ’why me,’ prompting them to engage in introspection regarding their experiences or potential causes of illness.

"*I am now starting to reflect on why I got this disease. In the past, when I looked at others, including myself, I could always see others’ faults and rarely see others’ strengths. Moreover, I felt I was not gentle enough towards this world when I reflected on myself. Firstly, I had a hot temper, and secondly, I rarely had the dependence and gentleness of women. I am not very gentle with my lover and children, and I belong to the type of person who is particularly dominant.”* [26]

"*I never imagined this would happen to me*, *so I have never bought critical illness insurance*. *Only after falling ill did I reflect on why it had to be me*. *Eventually*, *I realized that I hadn’t been loving myself enough*.*"* [28]

Reflective behavior serves as the primary ideological underpinning of PTG, prompting patients to reflect on their past actions in the context of illness, thereby facilitating self-cognition renewal. They progressively recognized their limitations and vulnerabilities.

“*Before my diagnosis, I just wanted to …… conquer the world. After diagnosis, I realised that I couldn’t do many of the things I wanted. I know me better, knowing what my limit is, knowing what I can do, and what I cannot do.”* [36]

It is noteworthy that renewal in self-cognition has the potential to facilitate patients’ reassessment and contemplation of life and mortality.

*3*.*5*.*1*.*2Category2*: *The enhancement of personal strength*. BC patients frequently exhibit an enhancement of personal strength following their diagnosis. The diagnostic process further catalyzes their potential, thereby augmenting their intrinsic power and resilience.

"*After getting this illness, I realized that no matter how much others try to comfort you, it’s pointless. The only thing that makes a difference is making peace with yourself."*(34)

"*I feel my tolerance increased after experience of disease*. *I think I can tolerate any event that occurs for me*.*"* [33]

" *After falling ill*, *I also engaged in self-reflection*. *The ailment bestowed diverse setbacks and agonies upon me*, *yet it was indeed a present from life*. *I am currently leading a life with greater purposefulness and a more pronounced sense of worth compared to the past*.*"* [28]

Furthermore, in the battle against illnesses, certain patients may develop a sense of adversarial engagement with their condition, thereby bolstering their confidence and equipping them to confront diverse challenges with an enhanced positive attitude toward life.

*"Everything needs to be viewed from two perspectives. This illness is a disaster for me, but I will not give up."* [29]

Enhancing inner strength and self-confidence facilitated a reflective process regarding past experiences, culminating in an awareness of their neglect of self-care, subjective well-being, and physical health. This prompts contemplation regarding their positioning and significance. They realize that personal happiness is paramount, as it is only through achieving one’s happiness that one can spread happiness to others.

"*I am increasingly focused on my thoughts and less concerned with others’ opinions. For instance, I used to worry about whether the gifts I bought for my relatives would be liked or judged, but now I believe that what matters most is that I feel it’s appropriate. At this stage in life, both parents and children need care. As an only child of my parents, I’ve realized the importance of taking care of myself first to care for my children, husband, and parents."* [26]

Compared to their pre-illness state, patients post-diagnosis also exhibit a sustained capacity for introspection regarding their illness experience, demonstrating acceptance, gratitude, and validation of their physical appearance, mental well-being, personality attributes, and behavioral patterns.

"*The doctor said I’m doing great with my recovery because I’ve been focused on watching what I eat, staying active. He even wants me to lead a health class! Honestly, I think I’ve been doing a pretty solid job taking care of myself despite being a cancer patient. My lifestyle is healthier than many others.”* [26]

"*I deem myself to be more confident and courageous than previously*. *If the illness has instructed me to cherish my present life*, *breast reconstruction has rendered me even more self-assured and attractive*. *"* [28]

*3*.*5*.*1*.*3Category3*: *Engage in proactive self-management of health conditions*. The self-management of illness encompasses three key components: self-monitoring, proactive acquisition of health-related information, and formulating management strategies. Following a diagnosis of breast cancer, patients develop an enhanced understanding of their bodies and adopt a proactive approach to health monitoring aimed at preventing recurrence and bolstering resilience against deterioration. They engage in reflective practices concerning their prior unhealthy lifestyle choices, establishing links between these behaviors and health promotion. Furthermore, they actively seek knowledge about the disease to foster an elevated health awareness.

"*I value my health maintenance and enjoy tuning into daily lectures on television and online platforms, such as Baiyibaishun, Beijing TV’s Famous Doctors Lecture Hall, and Health Diary. I like jotting down helpful methods in a notebook for later easy reference."* [26]

BC patients are inclined to enhance their health by developing structured exercise regimens or actively engaging in treatment.

"*While I once enjoyed consuming meat and considered it indispensable, I am now gradually acclimating to a diet of millet porridge and vegetables. I used to disregard my dietary habits, but I now recognize the significance of proper hydration. I consistently ensure I have a glass of water in hand in the morning and evening. I frequented the rehabilitation room twice weekly for exercise during the first half of the year. However, due to escalating work commitments and childcare responsibilities this year, my attendance has been limited to once a week. However, I will persist in engaging in a 30-minute daily walk within the confines of my workplace courtyard without experiencing any signs of fatigue."* [26]

"*Because my treatment was completed*, *I went to see a doctor who applied holistic medicine and then I took Chinese medicine*. *After two months*, *I felt my body get warmer*. *I heard BC patients should keep their bodies warm*, *so it [the Chinese medicine] is good for my body*.*"* [32]

Furthermore, they are increasingly recognizing the significance of emotional and stress management.

"*Now I’m gonna do my best to keep my emotions in check and stay positive and calm, or at least not let myself get too down. I used to just suck it up when things bothered me and try to comfort myself, but now I’m all about expressing how I feel. If something gets to me, I’ve gotta find a way to let it out. Maybe take a walk or just chill so my head can process things without getting all worked up. Overall, I’ve gotten way better at handling my feelings than before."* [26]

Some individuals may manage stress by refraining from engaging in stress-induced activities.

*"I didn’t use to say no, but now I can. I simply refuse to do anything which causes me to feel stressed out or I don’t want to do."* [35]

*3*.*5*.*1*.*4Category4*: *Always remember to be thankful*. Following a cancer diagnosis, patients often find themselves embracing a profound sense of gratitude towards their cherished family members, friends, fellow BC patients, healthcare professionals, and even the simple pleasures of life. This sentiment holds particular significance for cancer patients due to the intricate nature of their illness experience and the myriad forms of support they receive from loved ones and caregivers throughout their treatment journey.

*"The power of family unity and support from my friends, uh, friends who were concerned about me helped me out… er, I felt grateful that I was surviving thanks to the support from people around me… My family and friends, well, owing to them, I am here, I think."* [32]

Due to the heightened longing for ’life’ throughout the illness, patients, particularly those in advanced stages of the disease, will increasingly value each day and exhibit greater gratitude for their circumstances.

"*Living with this illness means taking each day as it comes and treasuring every moment. My family, particularly my brother, has provided unwavering support, consistently encouraging me to engage in physical activity and maintain a positive mindset. Subsequently, my emotional state improved as I redirected my focus. I endeavored to motivate myself to actively pursue treatment and adhere to a regular exercise regimen."* [26]

#### 3.5.2 Synthesized finding2: Enhancement of spirituality and religious belief

The cancer journey prompts each survivor to reflect on the spiritual dimensions of life, resulting in a novel understanding and appreciation of life’s meaning that diverges from their pre-diagnosis perspective. For patients with religious convictions, the experience of illness may heighten their reliance on faith as a source of spiritual strength.

*3*.*5*.*2*.*1Category5*: *Enhancing the inner self to facilitate spiritual development*. The spiritual growth experienced by BC patients who have undergone PTG is reflected in a novel understanding of life’s meaning. Throughout their illness, they reevaluate the significance of life, recognizing its inherent fragility and the inevitability of death.

"*I understand the value of life along with my spouse and children. Also I perceived health is valuable and we should appreciate it."* [33]

The impetus to overcome life-threatening illnesses compels breast cancer patients to cherish and appreciate life more profoundly. "Embracing life with affection, embracing the present" is the most authentic depiction of a patient’s attitude toward life’s significance.

"*I now truly relate to a saying: One doesn’t know to cherish until it’s lost. After going through this illness, my most profound perception is to cherish the people around me and life."* [36]

"*It is commonly asserted that "false alarms*, *restorations*, *and reunions following protracted separations" constitute the three significant felicities in life*. *We have no idea what awaits us tomorrow; thus*, *I highly value my present life*. *"* [27]

*3*.*5*.*2*.*2Category6*: *Reinforce one’s religious convictions*. Numerous patients with religious affiliations demonstrate an intensified focus on spiritual pursuits following the onset of illness. They report a significant enhancement in their spiritual resilience during this period, alongside a deepening connection to their faith. Patients from diverse cultural backgrounds may exhibit varying forms of religion-related growth.

"*After I had the disease, my faith in Jesus Christ became stronger. I now stopped pursuing those meaningless things. I just relied on Jesus to guide me in my life. I have peace in heart without any fear or confusion."* [36]

"*God (Allah) warned me not to get upset about anything and to prioritize my own needs*.*"* [35]

Certain patients hold the belief that employing religious content to elucidate their experiences with cancer can make them stronger.

"*I thought this disease was a test given by God. I stayed patient and grew stronger."* [35]

Some patients who did not hold religious beliefs prior to falling ill sought spiritual support and endeavored to cultivate new religious beliefs following their illness.

"*I was a non-religious before illness. My husband and my younger sister are Buddhists. I became a Buddhist after I got the disease. I often went to those worship ceremonies with them. Religion sometimes can bring us hope."* [36]

#### 3.5.3 Synthesized finding3: Appreciation of the new philosophy of life

BC patients undergo a significant transformation upon recognizing the complexities of their condition, which compels them to reevaluate the meaning of life and cultivate a deeper understanding. This transformative process engenders a rebirth, enabling them to attain an elevated state of existence.

*3*.*5*.*3*.*1Category7*: *Identify new opportunities and potential possibilities*. New opportunities and possibilities can be understood as patients recognizing transformative changes in their lives and embracing the desire to embark on a new chapter. Through active engagement in all facets of their treatment, patients gradually cultivate hope for the future and are eager to embrace novel challenges.

"*after the disease, I devised a new plan for my life, and tried to do things I hadn’t done before. I was successful, too. For example, I got my driving license, I passed a jewelry course. Now I am thinking of going to university."* [31]

"*In the aftermath of the surgery*, *I lost my breasts*, *yet fortunately*, *I persisted in survival*. *Consequently*, *notwithstanding the excruciating pain*, *there was concurrently a sensation of alleviation*. *I am resolute in maximizing the exploitation of my life hereafter and leading an extremely fulfilling existence*. *"* [29]

BC patients are prepared to advance into the future and possess a sense of hope and concrete plans.

"I got a qualification as a care manager [someone who develops a care plan for older people]… As I got it, I am thinking of working with elderly people and providing care in the future.*"* [32]

"*After I leave the hospital*, *I’m going to find things I love doing to help me heal and pick up some new hobbies*. *I’ll finally do everything I didn’t have time for and learn what I missed*. *This will make my days more fulfilling*, *and my mood will brighten up*, *too*.*"* [28]

*3*.*5*.*3*.*2Category8*: *The life priorities have transformed*. Following their confrontation with life and death, BC patients begin to reassess the significant aspects of existence and realize that "survival is the foundation for possessing everything," with health paramount. Following their perceived importance, they systematically prioritize elements such as family, career, financial stability, physical well-being, and other pursuits. Issues related to health and family life are regarded as critical.

"*After I fell ill, my lover has been constantly by my side without expressing any resentment towards me. What else could I possibly be dissatisfied with? I ought to make the fullest use of the remainder of my life. Health holds the utmost significance."* [36]

Patients in the postoperative recovery phase contemplate how to actualize the significance of their existence while actively engaging in treatment protocols aimed at restoring their well-being.

"*I plan to focus on getting healthier and practicing my rehabilitation exercises first. Then, once I’m feeling better physically, I’ll look for a job and start a small business. Due to my relatively young age, I cannot remain idle at home. It not only adds stress to the family but also feels really lonely being at home alone."* [26]

Some women reflect on their family life and realize that prioritizing their own needs and well-being is of paramount importance.

"*First, I meet my needs and then needs of others. I try doing things for myself. I used to take account of others’ criticisms, but now they are unimportant for me."* [35]

#### 3.5.4 Synthesized finding4: Transformations associated with others

When breast cancer patients confront the realities of life and death, they gain a deeper appreciation for the significance of their family and friends. They actively cherish those close to them, distance themselves from social relationships that induce discomfort, and enhance their empathy toward other cancer patients. This transformation has the potential to cultivate altruistic behavior and facilitate the reconstruction of individuals’ lives.

*3*.*5*.*4*.*1Category9*: *Proactively engage in establishing connections with others*. Prior to their illness, patients may have taken various familial relationships for granted; however, following the trauma, they reassess the significance of their partners, family members, friends, and other relational figures, leading them to actively cherish these connections.

"*I used to hold the opinion that my husband exhibited numerous problems in handling matters; however, I am currently commencing to observe some positive alterations. When we first got married, he was unable to wash his own socks, but now he looks after me and takes responsibility for the cooking and cleaning."* [26]

"*Initially*, *I deemed myself extremely unfortunate*. *I failed to have a satisfactory marriage during my youth*, *and now I’m afflicted with this disease*. *Nevertheless*, *my partner treats me exceptionally well*. *Although I have cancer at present*, *fortunately*, *it has not yet metastasized*. *I will value the remainder of my life*. *"* [34]

Furthermore, they actively engage in the establishment of intimate relationships.

"*After knowing I got the disease, I was closer with my family, children and friends. I cherish the time with them."* [36]

"*I’ve been getting closer to my friends*. *Initially*, *my friends contact with me; nowadays*, *I also actively invite them to join me for hiking*, *shopping*, *and watching movies*.*"* [27]

*"After completing chemotherapy and radiation therapy*, *I had wanted to learn how to paint at home*, *but now I want to improve my communication skills and connect with people better*. *I shouldn’t isolate myself by writing and painting alone in my world anymore*.*"*[26]

*3*.*5*.*4*.*2 Category10*: *Modification of interpersonal relationships*. Owing to the illness, patients inevitably face changes in their interpersonal relationships, although such changes may not always correspond with their subjective preferences.

"*I sold all valuable things after illness, with that I lost my relationship. My boyfriend decided to leave me. It is all material stuff, and that is ok for me. I learned who my good friends are. "* [36]

The experience of illness can facilitate patients in reevaluating their past interpersonal relationships, enabling them to make choices that align with their current needs.

"*I became aware of my values such as respect. Now I’ve limited my relationships with friends who do not respect me. I’ve become aware of the value of my friends who gave both financial and social support during diagnosis and treatment of the disease. It appears that the real gain in life is to have real friends."* [35]

Experiencing cancer may evoke feelings of stigma, particularly in specific cultural contexts where the topic is considered taboo and conversations about it are actively avoided. Consequently, some individuals facing this situation may create distance in their interpersonal relationships.

"*I usually refused to go to the parties organised by the factory I previously worked for. I did not feel I had a decent life as I did not own a house and I had a disease. That is why I was unwilling to have too much contact with my colleagues. They would just brag about the excellence of their husbands and kids. I did not want to compare with those that are better than me."* [36]

*3*.*5*.*4*.*3Category11*:*By assisting others through individual actions*. Following a diagnosis of breast cancer, patients demonstrate increased empathy towards others, which fosters altruistic behavior. They are capable of empathizing with fellow breast cancer patients and comprehending their experiences. Patients often share their narratives with others and offer support when necessary, thereby not only assisting them in reconstructing their disrupted values and worldview but also recognizing the intrinsic value of their own lives.

"*Previously, I held an aversion to the sentiment arising from the inability to furnish assistance. Consequently, I was prone to restrain myself or extend help when he urgently required it. I opine that offering aid to others is inherently a highly gratifying affair. After extending assistance, I discern that there subsists a sense of satisfaction and consolation within my heart. I particularly savor that sensation. Now, irrespective of whether it is successful, as long as I can assist others, I will exert every effort."* [28]

"*When I share my experience of illness with fellow patients*, *I am consistently eager and willing to do so*. *Following my health challenges*, *I have become even more resolute in aiding others*, *and the ability to offer assistance also provides me with a profound sense of fulfillment*.*"* [28]

This altruistic behavior is not only directed towards individuals with breast cancer of the same gender, but they also actively engage in other philanthropic and community-based public welfare activities to contribute to society.

"*As a member of the Anti-Cancer Association, I am pleased to provide guidance and support to fellow women, this presents an opportunity for me to contribute.”* [27]

“*leaving my body to science*.*"*[36]

## 4. Discussion

This meta-analysis synthesized 11 qualitative studies focusing on PTG in BC patients.

Following a meticulous re-examination of the primary research, four overarching themes of growth emerged: self-reconstruction, enhancement of spirituality and religious beliefs, appreciation of the new philosophy of life, and transformations associated with others.

### 4.1 Promote deliberate rumination

Significant gender differences in PTG have been observed [17], with one potential mediator being the greater inclination of women to engage in rumination compared to men [37]. Women demonstrate a greater tendency toward deliberate and brooding rumination [16]. The inclination to reflect on constructive aspects, such as an enhanced awareness of personal strengths and the recognition of the significance of social relationships, is regarded as a mechanism leading to greater reports of PTG [38]. Previous research has indicated that patients demonstrate self-reflective behaviors during their experiences of post-traumatic growth, engaging in reflection on their illness experiences to facilitate the refinement of self-awareness and the cultivation of new perspectives on mortality and existence. The PTG theory [39] posits that deliberate rumination of the cues from traumatic events can facilitate individuals in cultivating a positive perspective on the significance of the trauma, motivating them to reconstruct and contribute to the attainment of PTG. Deliberative rumination denotes the conscious process through which an individual systematically reviews and reevaluates events and associated stimuli [40]. Deliberate rumination plays a pivotal role in facilitating the transition of cancer patients from adverse psychological reactions to positive psychological transformations, aiding individuals in comprehending the post-traumatic world and discovering constructive significance in their traumatic experiences, thereby fostering the achievement of PTG and exerting a positive predictive influence on PTG [41]. Young breast cancer survivors exhibit more significant cancer-related impacts, heightened emotional distress, and poorer psychological adjustment compared to middle-aged or older women [42]. However, middle-aged patients demonstrate a higher receptivity to novel experiences and a quicker ability to break free from fixed thinking patterns than elderly patients [26]. They also excel in recognizing and comprehending traumatic events from multiple perspectives, thereby updating their cognitive systems—a favorable condition for the emergence and development of PTG among middle-aged breast cancer postoperative patients. Healthcare professionals should accurately comprehend the concept and mechanism of deliberate rumination and carefully monitor the level of deliberate rumination in breast cancer patients. They ought to promptly evaluate the type and characteristics of rumination and develop tailored psychological interventions based on the patient’s psychological state and age to enhance their adaptive cognitive processing level. Enhance avenues for patients to communicate with their families, healthcare professionals, or fellow cancer patients. Facilitate deliberate rumination opportunities for them. Healthcare professionals can help patients cultivate a constructive perception and mindset toward their illness through proactive language and demeanor. They assist patients in identifying specific objectives and resolutions, empowering them to confront their illness and treatment more effectively. This may foster the realization of PTG.

### 4.2 Facilitate emotional release

Research and theory suggest that PTG is unlikely to be manifested immediately after a significant event occurs but requires some time for its development and is more prone to be reported retrospectively [39,43,44]. The duration of illness is crucial in moderating positive and negative psychological health. From a short-term perspective, PTG is more strongly linked to adverse psychological health. However, its association with positive psychological health also becomes more robust over time, in accordance with the findings of Helgeson et al [12]. These research findings indicate that post-traumatic psychological interventions have a moderate short-term effect on reducing negative symptoms while demonstrating greater long-term efficacy in improving positive mental health outcomes. This aligns with the PTG functional description model proposed by Tedeschi and Calhoun [39,43], which emphasizes the importance of managing emotional distress during the initial stages of trauma. The participants enrolled in the study actively acquired skills to regulate and channel their emotions, irrespective of the stage of their diagnosis. Effective management of one’s emotional state is imperative for patients, indicating that healthcare professionals should offer tailored emotional support corresponding to the patient’s diagnosis. During the initial phases of disease diagnosis, engaging in discussions with the patient regarding their family or close relatives is crucial. This facilitates a comprehensive understanding of their role within the family unit and fosters a sense of purpose, motivating them to strive for recovery. It is advisable to assist the patient in establishing realistic self-assessment criteria and harnessing any positive emotions that may remain following the trauma. Simultaneously, it is vital to explore the patient’s inner world in order to promptly address and redirect negative emotions, promote constructive outlets for emotional release, guide attention toward meaningful aspects of life, and facilitate recognition of personal growth. Healthcare professionals should remain attuned to the emergence of positive attitudes in patients and promptly provide affirmation to facilitate the cultivation of their inherent growth potential. In the advanced phases of disease diagnosis, it is essential to maintain regular communication with patients to address their emotional needs, including self-expression, seeking assistance, and receiving validation. This approach can help mitigate negative emotions, focus on positive developments in the patient’s condition, recognize their progress, underscore individual agency, promote engagement in peer education initiatives, and foster a sense of purpose and fulfillment among patients. Ultimately, this contributes to achieving their life goals and facilitating personal growth.

### 4.3 Support effective coping strategies

The study involved participants with diverse coping styles, and it revealed that coping with trauma was significantly associated with post-traumatic growth. Specifically, religious coping, reappraisal coping, acceptance coping, and seeking support coping were all found to have significant positive correlations with participants’ PTG scores [45]. Coping mechanisms are intricately linked to cultural backgrounds, and culture profoundly influences individuals’ behaviors and their comprehension and interpretation of others’ behaviors [46]. Familiarity with an individual’s cultural background and values can significantly enhance the comprehension of their attitudes and responses toward traumatic events [46]. Specific cultural factors substantially influence women’s articulation of their illnesses, interpretation of their body images, pursuit of existential meaning, and adoption of coping strategies. For instance, within the context of Chinese culture, the utilization of the "non-action" coping strategy, emphasis on family-centered approaches, and adoption of Chinese-style self-disclosure can collectively contribute to the adaptation process and PTG experience among Chinese female BC survivors [47]. Religious beliefs also influence coping mechanisms, and research indicates that spiritual beliefs play a significant role in PTG, contributing to an enhanced overall quality of life [34]. Religious coping pertains to an individual’s ability to utilize religious beliefs and practices for comprehending and managing stress in everyday life and during emergencies. It serves multiple functions in both routine existence and periods of upheaval: attributing significance to adverse occurrences, providing a framework for attaining a sense of mastery over challenging circumstances, offering solace during trying times, fostering close relationships within religious communities, and aiding individuals in navigating significant life transitions [48]. Religion coping has a more significant positive impact on the elderly and women [45], and there exists a reciprocal relationship between religiosity and PTG [40], whereby stronger religious beliefs enhance a sense of control and a propensity to seek meaning [5]. Psychological support constitutes a vital component of cancer care. Research indicates that Mindfulness-Based Stress Reduction (MBSR) can enhance mindfulness and decrease avoidance of distressing thoughts, images, emotions, and physical sensations, thereby facilitating post-traumatic growth [49]. Moreover, healthcare professionals should develop culturally and religiously tailored interventions for patients from diverse racial and cultural backgrounds to facilitate effective coping with their illnesses. It is feasible to encourage patients with religious beliefs to utilize religious connotations for interpreting their experiences of illness, seeking new perspectives during and after trauma, and developing novel life philosophies.

### 4.4 Enhance social support systems

The breast cancer patients included in the study generally reported enhanced social relationships. Prior research has unveiled a positive correlation between perceived social support and PTG [50]. Female, older, unemployed, and less educated vulnerable survivors tend to report notably lower levels of PTG [51]. Support from spouses, family members, and fellow survivors assumes a crucial role in the process of PTG [34]. Social support may facilitate PTG by mitigating individuals’ stress appraisals [52]. Following the social cognitive processing model [53], the presence of unsupportive social networks increases the probability of developing PTSD while also impeding personal growth. Other research also indicates that individuals of Chinese or Asian descent may derive greater benefit from covert social (individuals can derive emotional solace from social networks without the need to divulge or deliberate on their issues or stressors) support as opposed to overt social support(individuals actively seek and leverage social networks to access support(individuals actively seek and leverage social networks to access support) [54].

During the initial phase of treatment, healthcare professionals must provide essential support and assistance. This includes delivering specialized health education, facilitating postoperative rehabilitation, and imparting pertinent knowledge and skills. Moreover, they should promptly address patients’ physical, emotional, and postoperative recovery challenges while offering comprehensive guidance through this transitional period. As patients gradually reintegrate into work and family life, substantive and informational support’s significance becomes increasingly pronounced. The sense of responsibility towards loved ones is a crucial motivator for patients to persevere and combat the disease. Their encouragement and companionship often catalyze the development of new positive cognitive patterns. Healthcare professionals can facilitate collective education for family members of BC patients, guide patients in adopting a health-conscious lifestyle conducive to reintegration into their familial and societal roles, mitigate potential complications, enhance the quality of life, offer substantial support to patients and their families in coping with the emotional distress they have undergone, and promote cohesive familial dynamics through increased interaction and communication. The support from fellow survivors during the later recovery phase is crucial. Morris et al. [55] asserted that the peer support program establishes a secure social milieu, which is beneficial for enhancing comprehension and acceptance, facilitating self-exploration, and thereby facilitating PTG through active participation in the peer support program [56], where female participants received support from their companions. This support facilitated their transition from identifying as cancer patients to embracing an active survivor identity, leading to positive alterations in self-perception, interpersonal relations, and life philosophy, thereby fostering PTG. Healthcare professionals can facilitate the establishment of online platforms, mutual aid associations, and support groups to enable BC patients to establish communication channels with peers, fostering mutual exchange and assistance among them. This will contribute to the robust development of patients’ social support systems.

## 5. The review’s strengths and limitations

This study rigorously adhered to the JBI-Meta integration method and the quality evaluation standards for qualitative research established by JBI in order to conduct a qualitative evidence synthesis on the PTG experience of BC patients. Nevertheless, this study undoubtedly has certain restrictions. Firstly, the final inclusion comprised only 11 articles, indicating a restricted scope of coverage. All selected studies were conducted in Asian and European countries. The differing cultural backgrounds, economic levels, medical standards, and policies across these regions may restrict the universality of the research results. Consequently, the conclusions drawn from this review can merely be extrapolated to analogous cultural and medical technology contexts. Secondly, in light of the dearth of relevant studies on the PTG experience of male breast cancer patients, all the study subjects encompassed in the review were female. Furthermore, the methodological rigor of the studies encompassed in the review was appraised as being moderate, potentially impacting the overall reliability of the study findings. Moreover, most of the literature lacked an explicit introduction to researchers’ cultural and values perspectives, which could introduce certain biases. Ultimately, the study participants were at various stages of their cancer journey and underwent diverse treatments. The duration between a patient’s cancer diagnosis and their involvement in the study could potentially impact their capacity to manage the trauma, subsequently influencing the degree of trauma-related stress or growth experienced. When a patient undergoes diagnosis, surgery, follow-up treatment, the transition to the survival period, the survival period itself, recurrence, and remission, each novel trauma along the trajectory of cancer may precipitate multiple alterations in this relationship [57]. Hence, we might be incapable of comprehensively capturing the PTG experiences of BC patients of diverse genders at various stages and undergoing disparate treatment modalities. Given that meta-analysis relies on the analysis of incorporated studies, the reliability of synthesized results is inherently constrained by the quality of the original studies. In the future, research would benefit from more high-quality original studies, particularly those that incorporate theoretical frameworks within cultural contexts.

## 6. Conclusions

This study has synthesized qualitative evidence regarding the experiences of PTG in BC patients, thereby contributing to an increased awareness of such experiences from the patient’s perspective. It is essential to comprehend the psychological and social changes experienced by patients and integrate them into management strategies. The findings indicate that BC patients exhibit positive changes in personal, internal, interpersonal, and health-related behavioral aspects during their post-traumatic growth experience. It is recommended that healthcare professionals and researchers focus on the psychological well-being and positive transformations of BC patients. They should prioritize the utilization of positive psychological intervention methods, encourage deliberate rumination, provide emotional guidance and effective coping strategies, facilitate improved family support, assist in establishing peer support systems, promote post-traumatic growth in patients, and simultaneously aid patients in adjusting to their new family dynamics and daily routines through comprehensive health guidance. A comprehensive support system, integrating the forces of the family, the hospital, and the society, should be constructed to offer prompt solutions and meet the needs of patients throughout their treatment process. Furthermore, practical interventions ought to be developed to enhance the quality of life for patients.

## Supporting information

S1 File PRISMA-P (Preferred Reporting Items for Systematic review and Meta-Analysis Protocols) 2020 checklist for this systematic review. (DOCX)

S2 File ENTREQ checklist (enhancing transparency in reporting the synthesis of qualitative research). (DOCX)

S3 File Search strategy on February 28, 2024. (DOCX)

S4 File JoannaBriggsInstitute5-stage approach to meta-aggregation. (DOCX)

S5 File Data availability statement. (DOCX)

S6 File Identified studies. (DOCX)
