# Supplementary material for: Post-traumatic growth experience of breast cancer patients: A qualitative systematic review and meta-synthesis
Source: PLoS One. 2025 Jan 23;20(1):e0316108. doi: 10.1371/journal.pone.0316108 (PMC11756777; doi:10.1371/journal.pone.0316108)
Supplement: S2 File — (DOCX) [file pone.0316108.s002.docx]

| No. Item | Guide Questions/Description | Reported on Page |
| --- | --- | --- |
| 1. Aim | The objective of this article is to synthesize the existing evidence in order to gain insight into the experiences of post-traumatic growth among individuals diagnosed with breast cancer. | P1 |
| 2. Synthesis methodology | Identify the synthesis methodology or theoretical framework that underpins the synthesis, and describe the rationale for the choice of methodology (e.g., meta-ethnography, thematic synthesis, critical interpretive synthesis, grounded theory synthesis, realist synthesis, meta-aggregation, meta-study, framework synthesis) | P5 |
| 3. Approach to searching | The search was pre-planned. Comprehensive search strategies were undertaken to seek all available studies. | P6;Supplementary file-3 |
| 4. Inclusion criteria | The included studies used widely accepted qualitative data collection methods with well-described methodology, including interviews, focus groups, direct observation, and participatory action research. They also needed to clearly describe recognized qualitative data analysis methods (e.g., grounded theory, narrative analysis, content analysis, thematic analysis). Excluded studies included those for which it took much work to extract qualitative data, including mixed-methods studies lacking delineated data and those authored in languages other than Chinese or English. Commentaries, protocols, and systematic reviews were not included in the analysis. | P6-7 |
| 5. Data sources | We systematically query four Chinese databases, including China Biomedical Literature Database (CBM), WanFang, China National Knowledge Infrastructure (CNKI), and VIP, alongside seven English databases such as PubMed, Embase, CINAHL, PsyINFO, Web of Science, SCOPUS, and Cochrane Library to comprehensively retrieve qualitative studies focusing on the PTG experiences of breast cancer patients. The search encompasses data from the inception of these databases up to February 2024. Additionally, a thorough examination of the references cited in the included studies is conducted to supplement the acquisition of pertinent literature. The search strategy incorporates terms such as "breast neoplasms,"、" breast cancer,"、" post-traumatic growth"、" post-traumatic growth, "、" post-traumatic growth,"、"pig,"、" qualitative research,"、" qualitative study, "、" phenomenology,∗" and so on. Searching strategies in the database can be found in the supplementary file 3. | P6 |
| 6. Electronic Search strategy | Supplementary file 3 describes the literature search | Supplementary file-3 |
| 7. Study screening methods | We used Endnote 20 software to screen and analyze the literature by importing all citations and removing duplicates. Two reviewers independently screened articles based on title and abstract information, while a third reviewer settled any divergences. A second round of review was conducted for articles meeting the inclusion criteria using a process similar to the first round. All references to the included articles were searched for additional potentially relevant studies (see Figure 1). | P7 |
| 8. Study characteristics | Table 2 presents the characteristics of the included studies (author(s), year of publication, country, population, number of participants, data collection, methodology, and analysis ). | Table 2 |
| 9. Study selection results | Figure 1 presents a flow diagram using PRISMA guidelines for reporting systematic reviews, which shows the selection process and results. | Figure 1 |
| 10. Rationale for appraisal | The process involved two reviewers who independently assessed the quality of the included studies using the "Quality Appraisal Standards for Qualitative Research" from the Australian Centre for Evidence-Based Healthcare (JBI). Both reviewers had received training in evidence-based practice methodology. Each criterion was assessed with responses of "yes," "no," "unclear," or "not applicable." The incorporated studies were sorted into three levels: A, B, and C. Studies that fully met the quality standards were designated as level A, suggesting the existence of a low risk of bias; those that partially met the standards were classified as level B, suggesting the existence of a moderate risk of bias; and those that did not meet the standards were classified as level C, suggesting the existence of a high risk of bias. In cases where there was disagreement between reviewers' assessments, a third reviewer provided input to facilitate consensus. Ultimately, only studies rated at levels A and B in terms of quality were included. | P8 |
| 11. Appraisal items | Checklist for Qualitative Research (Critical Appraisal tools for use in JBI Systematic Reviews)，ConQual system scores | P8-9; Table 1、Table 4 |
| 12.Appraisal process | Two independent reviewers conducted the appraisal and discussed whether consensus was required. When the evaluation results conflicted, the third researcher decided | P8-9 |
| 13. Appraisal results | Appraisal results are presented in Table 1 and Table 4 | Table 1、Table 4 |
| 14. Data extraction | Two reviewers were designated to extract data from both Chinese and English literature. The reviewer assigned to the Chinese literature translated the research findings into English, which the other two reviewers subsequently assessed to ensure that the translation accurately represented the original Chinese text. In instances of disagreement, they would consult a fourth reviewer until all reviewers reached a consensus. Two reviewers autonomously extracted data from the included studies utilizing standardized forms. In case of disagreement, they discussed the issue with a third reviewer. The basic features of the included studies, such as authors, year of publication, country, study design, study population and characteristics, interesting phenomena, and main outcomes, were extracted using standardized forms. | P7 |
| 15. Software | Database search results were first imported into the reference management software program Endnote 20. After duplicates were removed, titles and abstracts were read for the eligibility assessment. | P7 |
| 16. Number of reviewers | A minimum of two trained reviewers (SNH, MH) | P6-9 |
| 17. Coding | JBI meta-aggregation did not use the technique of coding | NA^＊＊^ |
| 18.Study comparison | The aggregation process involves synthesizing findings by categorizing them by their similarity in meaning. Then, we subject these categories to a synthesis to generate more comprehensive findings | P8-9 |
| 19. Derivation of themes | We subjected these categories to further synthesis to generate more comprehensive findings called synthesized findings. Only unequivocal and credible findings were included. Not supported findings were not presented in the synthesis or the results (Lockwood et al., 2015) | P8-10 |
| 20. Quotations | Provide quotations from the primary studies to illustrate themes/constructs, and identify whether the quotations were participant quotations of the author’s interpretation. | P16-26 |
| 21. Synthesis output | Present rich, compelling, and useful results that go beyond a summary of the primary studies | P27-33 |

^＊^Reference: Tong A, Flemming K, McInnes E, Oliver SA, Craig J. Enhancing transparency in reporting the synthesis of qualitative research: ENTREQ. BMC Medical Research Methodology 2012, 12:181.

＊＊NA means 'not applicable'
