# Supplementary material for: Post-traumatic growth experience of breast cancer patients: A qualitative systematic review and meta-synthesis
Source: PLoS One. 2025 Jan 23;20(1):e0316108. doi: 10.1371/journal.pone.0316108 (PMC11756777; doi:10.1371/journal.pone.0316108)
Supplement: S3 File — (DOCX) [file pone.0316108.s003.docx]

| Records retrieved | Query | No. |
| --- | --- | --- |
| 731153 | 'bilateral breast neoplasm'/exp OR 'bilateral breast neoplasm' OR 'bilateral breast tumor'/exp OR 'bilateral breast tumor' OR 'bilateral breast tumour'/exp OR 'bilateral breast tumour' OR 'breast gland tumor'/exp OR 'breast gland tumor' OR 'breast gland tumour'/exp OR 'breast gland tumour' OR 'breast mass'/exp OR 'breast mass' OR 'breast neoplasia'/exp OR 'breast neoplasia' OR 'breast neoplasm'/exp OR 'breast neoplasm' OR 'breast neoplasms'/exp OR 'breast neoplasms' OR 'breast neoplasms, male'/exp OR 'breast neoplasms, male' OR 'breast tumorigenesis'/exp OR 'breast tumorigenesis' OR 'breast tumour'/exp OR 'breast tumour' OR 'female breast neoplasm'/exp OR 'female breast neoplasm' OR 'female breast tumor'/exp OR 'female breast tumor' OR 'female breast tumour'/exp OR 'female breast tumour' OR 'male breast neoplasm'/exp OR 'male breast neoplasm' OR 'male breast tumor'/exp OR 'male breast tumor' OR 'male breast tumour'/exp OR 'male breast tumour' OR 'mamma tumor'/exp OR 'mamma tumor' OR 'mamma tumour'/exp OR 'mamma tumour' OR 'mammary gland neoplasia'/exp OR 'mammary gland neoplasia' OR 'mammary gland neoplasm'/exp OR 'mammary gland neoplasm' OR 'mammary gland tumor'/exp OR 'mammary gland tumor' OR 'mammary gland tumorigenesis'/exp OR 'mammary gland tumorigenesis' OR 'mammary gland tumour'/exp OR 'mammary gland tumour' OR 'mammary neoplasia'/exp OR 'mammary neoplasia' OR 'mammary neoplasm'/exp OR 'mammary neoplasm' OR 'mammary neoplasms'/exp OR 'mammary neoplasms' OR 'mammary tumor'/exp OR 'mammary tumor' OR 'mammary tumor cell'/exp OR 'mammary tumor cell' OR 'mammary tumorigenesis'/exp OR 'mammary tumorigenesis' OR 'mammary tumour'/exp OR 'mammary tumour' OR 'mammary tumour cell'/exp OR 'mammary tumour cell' OR 'mass in the breast'/exp OR 'mass in the breast' OR 'masses in the breast'/exp OR 'masses in the breast' OR 'neoplasia of the breast'/exp OR 'neoplasia of the breast' OR 'neoplasm of the breast'/exp OR 'neoplasm of the breast' OR 'neoplasm of the mammary gland'/exp OR 'neoplasm of the mammary gland' OR 'neoplastic breast'/exp OR 'neoplastic breast' OR 'neoplastic mammary'/exp OR 'neoplastic mammary' OR 'neoplastic mammary gland'/exp OR 'neoplastic mammary gland' OR 'tumor of the breast'/exp OR 'tumor of the breast' OR 'tumor of the female breast'/exp OR 'tumor of the female breast' OR 'tumor of the male breast'/exp OR 'tumor of the male breast' OR 'tumor of the mammary gland'/exp OR 'tumor of the mammary gland' OR 'tumorigenesis of the breast'/exp OR 'tumorigenesis of the breast' OR 'tumorigenesis of the mammary gland'/exp OR 'tumorigenesis of the mammary gland' OR 'tumour of the male breast'/exp OR 'tumour of the male breast' OR 'unilateral breast neoplasm'/exp OR 'unilateral breast neoplasm' OR 'unilateral breast neoplasms'/exp OR 'unilateral breast neoplasms' OR 'unilateral breast tumor'/exp OR 'unilateral breast tumor' OR 'breast tumor'/exp OR 'breast tumor' | #1 |
| 2883 | 'post-traumatic growth (psychology)'/exp OR 'post-traumatic growth (psychology)' OR 'post-traumatic psychological growth'/exp OR 'post-traumatic psychological growth' OR 'posttraumatic growth, psychological'/exp OR 'posttraumatic growth, psychological' OR 'posttraumatic psychological growth'/exp OR 'posttraumatic psychological growth' OR 'psychological growth following trauma'/exp OR 'psychological growth following trauma' OR 'psychological post-traumatic growth'/exp OR 'psychological post-traumatic growth' OR 'psychological posttraumatic growth'/exp OR 'psychological posttraumatic growth' OR 'posttraumatic growth (psychology)'/exp OR 'posttraumatic growth (psychology)' OR 'ptg' | #2 |
| 1436480 | ‘qualitative’ OR ‘mixed study’ OR ‘mixed research’ OR ‘mixed method’ OR ‘phenomenology∗’ OR ‘grounded theory’ OR ‘ethnography∗’ OR ‘case stud∗’ OR ‘action research’ OR ‘interview∗’ OR ‘focus group’ OR ‘observation∗’ | #3 |
| 20 | #1 AND #2 AND #3 | #4 |
