# Supplementary material for: Post-traumatic growth experience of breast cancer patients: A qualitative systematic review and meta-synthesis
Source: PLoS One. 2025 Jan 23;20(1):e0316108. doi: 10.1371/journal.pone.0316108 (PMC11756777; doi:10.1371/journal.pone.0316108)
Supplement: S4 File — (DOCX) [file pone.0316108.s004.docx]

| **Stage** | **Details** | **Evidence** |
| --- | --- | --- |
| 1 | Critical appraisal – All included studies were subject to critical appraisal prior to data extraction. This also included a review of the credibility and dependability of the studies included. | Joanna Briggs Institute Critical Appraisal Tool |
| 2 | Extraction – Study characteristics were extracted including study methods, phenomenon of interest, theme and sub themes (verbatim and illustrative quote). | Joanna Briggs Institute Qualitative Extraction Tool |
| 3 | Categorisation – The results of the studies (themes and sub-themes) were initially grouped and then given a categorisation. | Joanna Briggs Institute Meta-Aggregation Approach |
| 4 | Synthesised findings – The categories generated then lead to the creation of ‘synthesised findings’ that provide a comprehensive and representative conclusion to the categories. | Joanna Briggs Institute Meta-Aggregation Approach |
| 5 | Evidence level – Each study that forms parts of the synthesised finding was given a designated evidence levels of 1) unequivocal (open to challenge), 2) credible (interpretation) and 3) unsupported (no supporting evidence) – this relates to themes being supported by citations or interview excerpts provided by participants. This is then taken into account for the ConQual score regarding the confidence for each finding. |  |
