# Supplementary material for: Post-traumatic growth experience of breast cancer patients: A qualitative systematic review and meta-synthesis
Source: PLoS One. 2025 Jan 23;20(1):e0316108. doi: 10.1371/journal.pone.0316108 (PMC11756777; doi:10.1371/journal.pone.0316108)
Supplement: S5 File — (DOCX) [file pone.0316108.s005.docx]

**Data Availability statement**

All the data generated throughout this study are encompassed within this paper (and its supplementary file 8). Owing to copyright constraints, we are precluded from providing the complete text of the original literature incorporated in the analysis. Nevertheless, the data substantiating the study's findings can be accessed from the public databases delineated in the table.

**Database link:**

China National Knowledge Infrastructure (CNKI) : <https://www.cnki.net>

WanFang: https://www.wanfangdata.com.cn

PubMed: https://pubmed.ncbi.nlm.nih.gov

Web of Science: https://clarivate.com.cn/solutions/web-of-science/

CINAHL:https://web-p-ebscohost-com-s.webvpn.cams.tsgvip.top/ehost/search/advanced?vid=1&sid=7008d587-6746-491e-b1a0-461ae8458880%40redis

| Reference number | Author(s) &Publication Year | Database Name | Doi/URL | References link |
| --- | --- | --- | --- | --- |
| 30 | Horgan et al. (2010) | PubMed | 10.1002/pon.1825 | https://pubmed.ncbi.nlm.nih.gov/20734340/ |
| 29 | Li et al（2012） | WanFang | 10.3969/j.issn.1671-315X.2012.09.023 | <https://d.wanfangdata.com.cn/periodicalCh9QZXJpb2RpY2FsQ0hJTmV3UzIwMjQxMTA1MTcxMzA0Eg9obGdsenoyMDEyMDkwMjMaCHpybTMyMXZp> |
| 31 | Fallah et al. (2012) | CINAHL | https://web-p-ebscohost-com-s.webvpn.cams.tsgvip.top/ehost/search/advanced?vid=1&sid=7008d587-6746-491e-b1a0-461ae8458880%40redis | https://search-ebscohost-com-443.webvpn.cams.tsgvip.top/login.aspx?direct=true&db=cul&AN=85319799&lang=zh-cn&site=ehost-live |
| 32 | Tsuchiya et al. (2013) | PubMed | 10.1080/13548506.2012.686620 | https://pubmed.ncbi.nlm.nih.gov/22646702/ |
| 33 | Mehrabi et al. (2015) | PubMed | 10.14661/1239 | https://pubmed.ncbi.nlm.nih.gov/26435823/ |
| 34 | Barthakur et al. (2016) | PubMed | 10.4103/0973-1075.179609 | https://pubmed.ncbi.nlm.nih.gov/27162426/ |
| 26 | Tang et al. (2019) | CNKI | 10.16821/j.cnki.hsjx.2019.08.023 | https://kns.cnki.net/kcms2/article/abstract?v=QnUIp1GSfy7I3-M5F2mZQtZsWAt7LeLNSKtClYde3xAAcc_-UoN3GA6AO8J_R8F4eph3HwXj3sr0srLy9FbiiO0mF32le0xZkb1cQZ1F7SUbPsN-dNfPdJLGEUPEsbfGQPAnUSYrJPt1lGyrINQk4n_pOW3io8DP_4Z0EJ3G-Rf-Zvg_NMShCQpsH1dJUCzK&uniplatform=NZKPT&language=CHS |
| 35 | İnan et al. (2020) | PubMed | 10.5152/ejbh.2019.5006 | https://pubmed.ncbi.nlm.nih.gov/31912017/ |
| 36 | Zhai et al (2021) | PubMed | 10.1177/1049732320930195 | https://pubmed.ncbi.nlm.nih.gov/32564672/ |
| 27 | Tan et al (2023) | CNKI | 10.16460/j.issn1008-9969.2023.23.073 | https://kns.cnki.net/kcms2/article/abstract?v=QnUIp1GSfy5yTq_4Nglk1tKU-s7u6L8pRQMrjC2n9_FfUvfJW_ekHiWpbRhnDl8tnRhwVlw51cJ5T6heyx_CeZWv1dqwKlxOovNn81xhe0C_vXDGVWkvAOqDZgbLpV_KEOIX9dsWrchTrFFCbeD7MsvkeuDpwUlJ7NUSWGi1TDh7Y3RxO_rzJy2Hkfz-vP8a&uniplatform=NZKPT&language=CHS |
| 28 | Yan et al. (2023) | WanFang | 10.3870/j.issn.1001-4152.2023.17.100 | https://d.wanfangdata.com.cn/periodical/Ch9QZXJpb2RpY2FsQ0hJTmV3UzIwMjQxMTA1MTcxMzA0Eg5obHh6ejIwMjMxNzAyNRoIaXdka3MyNmY%3D |
