# Supplementary material for: Post-traumatic growth experience of breast cancer patients: A qualitative systematic review and meta-synthesis
Source: PLoS One. 2025 Jan 23;20(1):e0316108. doi: 10.1371/journal.pone.0316108 (PMC11756777; doi:10.1371/journal.pone.0316108)
Supplement: S6 File — (DOCX) [file pone.0316108.s006.docx]

| Author(s) &Publication Year | Research location | Name of data extractors/reviewers | Reason for exclusion | Reasons for inclusion | date of data extraction |
| --- | --- | --- | --- | --- | --- |
| **Horgan et al. (2010)** | England | ShiNi Huang | / | The research subjects and content meet the inclusion criteria, and it is possible to extract the available data and content. | 2024/3/10 |
| Li et al（2012） | China | Min Huang | / | The research subjects and content meet the inclusion criteria, and it is possible to extract the available data and content. | 2024/3/10 |
| Fallah et al. (2012) | Iran | ShiNi Huang | / | The research subjects and content meet the inclusion criteria, and it is possible to extract the available data and content. | 2024/3/10 |
| Cheng et al. (2016) | China | Min Huang | Not paying attention to the PTG experience | / | / |
| Yastibaş et al. (2022) | Turkey | ShiNi Huang | Irregular qualitative research design | / | / |
| Tsuchiya et al. (2013) | Japan | ShiNi Huang | / | The research subjects and content meet the inclusion criteria, and it is possible to extract the available data and content. | 2024/3/11 |
| Tang et al. (2019) | China | Min Huang | / | The research subjects and content meet the inclusion criteria, and it is possible to extract the available data and content. | 2024/3/11 |
| Gorven et al. (2018) | South Africa | ShiNi Huang | Unable to extract enough content | / | / |
| Zhu et al. (2020) | China | Min Huang | Irregular qualitative research design | / | / |
| Mehrabi et al. (2015) | Iran | ShiNi Huang | / | The research subjects and content meet the inclusion criteria, and it is possible to extract the available data and content. | 2024/3/11 |
| Zhai et al (2021) | China | ShiNi Huang | / | The research subjects and content meet the inclusion criteria, and it is possible to extract the available data and content. | 2024/3/12 |
| Zhai et al (2020) | China | ShiNi Huang | Unable to extract enough content | / | / |
| Burke et al. (2012) | Canada | Min Huang | Not paying attention to the PTG experience | / | / |
| ShamsUn et al. (2017) | India | Min Huang | Unable to extract enough content | / | / |
| Lelorain et al. (2012) | France | ShiNi Huang | Unable to extract enough content | / | / |
| Masood et al. (2023) | Pakistan | ShiNi Huang | Not paying attention to the PTG experience | / | / |
| Niebauer et al. (2021) | USA | Min Huang | Not paying attention to the PTG experience | / | / |
| Trisha et al. (2018) | USA | ShiNi Huang | Not paying attention to the PTG experience | / | / |
| Barthakur et al. (2016) | India | ShiNi Huang | / | The research subjects and content meet the inclusion criteria, and it is possible to extract the available data and content. | 2024/3/12 |
| Heras et al. (2013) | Greece | Min Huang | Not paying attention to the PTG experience | / | / |
| Ciria-Suarez et al. (2021) | Spain | ShiNi Huang | Not paying attention to the PTG experience | / | / |
| Lelorain et al. (2012) | UK | ShiNi Huang | Not paying attention to the PTG experience | / | / |
| Tan et al (2023) | China | Min Huang | / | The research subjects and content meet the inclusion criteria, and it is possible to extract the available data and content. | 2024/3/13 |
| İnan et al. (2020) | Turkey | ShiNi Huang | / | The research subjects and content meet the inclusion criteria, and it is possible to extract the available data and content. | 2024/3/13 |
| Kroemeke et al. (2017) | Poland | Min Huang | Unable to extract enough content | / | / |
| Canan et al. (2012) | Turkey | Min Huang | Unable to extract enough content | / | / |
| Andysz et al. (2016) | Poland | ShiNi Huang | Unable to extract enough content | / | / |
